# Effectiveness of multifaceted interventions including motivational interviewing and home-based rehabilitation program for improving mental and physical health in stroke patients: A randomized controlled trial

**DOI:** 10.1016/j.ijnsa.2024.100259

**Published:** 2024-10-30

**Authors:** Thao Thi Phuong Nguyen, Hai Bui Hoang, Huyen Thi Thanh Vu

**Affiliations:** a Institute for Preventive Medicine and Public Health, Hanoi Medical University, Hanoi 100000, Vietnam; b Academy of Medical Sciences, Ho Chi Minh 700000, Vietnam; c Department of Emergency and Critical Care Medicine, Hanoi Medical University, Hanoi 100000, Vietnam; d Emergency and Critical Care Department, Hanoi Medical University Hospital, Hanoi Medical University, Hanoi 100000, Vietnam; e Department of Geriatrics, Hanoi Medical University, Hanoi 100000, Vietnam; f Scientific Research Department, National Geriatric Hospital, Hanoi 100000, Vietnam

**Keywords:** Stroke, Depression, Fatigue, Cognitive impairments, Mental health, Rehabilitation

## Abstract

**Background:**

In Vietnam, early screening and intervention for post-stroke mental health are limited, with rising demand for home-based rehabilitation due to the scarcity of inpatient programs.

**Objectives:**

We aimed to evaluate the effectiveness of multifaceted interventions, including Motivational Interviewing and home-based rehabilitation, in improving the mental and physical health of stroke patients.

**Design:**

A randomized controlled trial at the Vietnam National Geriatric Hospital assessed a multi-intervention approach for stroke survivors from 2021 to 2022. Ninety-two participants (aged over 45 with a stroke diagnosis) were randomly assigned to an intervention group (Motivational Interviewing and home-based rehabilitation) or a control group (standard care), with 46 participants in each group. Outcomes for mental health (Patient Health Questionnaire-9, Fatigue Severity Scale, Mini-Mental State Examination) and physical health (Barthel Index) were measured at baseline and after 1, 3, and 6 months. Statistical analyses used *t*-tests, Cohen's *d*, and repeated measures ANOVA.

**Results:**

Over 6 months, 37 participants completed the intervention program. Baseline characteristics were similar except for age, lesion locations, and National Institutes of Health Stroke Scale scores. The intervention group showed significant improvements in mental health and physical function. Patient Health Questionnaire-9 scores decreased from 9.1 to 1.8, and Fatigue Severity Scale scores dropped from 28.5 to 17.8, while Barthel Index scores improved from 58.8 to 68.8 (*p* < 0.001).

**Conclusions:**

We found that participants who underwent Motivational Interviewing therapy and home-based rehabilitation were more likely than those receiving standard care to experience substantial improvements in both mental and physical health metrics.

**Registration:**

The research protocol was registered on ClinicalTrials.gov on August 1, 2021 (Identifier: NCT04941482, link: https://clinicaltrials.gov/study/NCT04941482).

**What is already known about the topic**

Post-stroke patients often face physical impairments, including decreased mobility and balance, as well as mental health issues such as depression, fatigue, and cognitive impairment.

Early screening and intervention for post-stroke mental health are limited, with rising demand for home-based rehabilitation due to the scarcity of inpatient programs in Vietnam.

What this paper adds

Participants who underwent Motivational Interviewing therapy and home-based rehabilitation were more likely to experience improved recovery and reduced depression and fatigue.

Home-based individualized exercises, supported by family involvement and audiovisual materials, accelerated recovery and improved functional independence.

Motivational Interviewing was effective in reducing depression and fatigue, enhancing the overall rehabilitation outcomes for stroke survivors.

Alt-text: Unlabelled box

## 1 Introduction

According to the World Health Organization, stroke is the second leading cause of mortality and the third highest contributor to disabilities (Feigin et al., 2017). In the Southeast Asia region, stroke prevalence varies: Singapore at 36.5 (over 45 years old), Thailand at 18.8 (over 45 years old), the Philippines at 9.0, Indonesia at 8.0, Malaysia at 7.0, and Vietnam at 6.1 per 1000 population (Ahmad Ainuddin et al., 2021). Stroke poses a significant public health concern, as it is the primary cause of both mental and physical health impairments among adults (Donkor, 2018).

A previous systematic review revealed that 40 % of stroke survivors experienced physical disabilities requiring specialized treatment, while an additional 10 % required care in nursing homes or other long-term rehabilitation centers (Aldehaim et al., 2016). Post-stroke physical impairment was associated with decreased mobility, aerobic fitness, and balance, as well as a worsened quality of life (Field et al., 2013). Additionally, post-stroke mental health issues, including depression, fatigue, and cognitive impairment, were recognized as significantly influencing rehabilitation outcomes and reducing quality of life (Plastow et al., 2021). Approximately 30 % of individuals who experienced a stroke develop depressive symptoms that extend beyond typical reactions to their current health status (Hackett and Pickles, 2014). Previous researchers indicated that being diagnosed with post-stroke depression is related to a decline in both quality of life and life expectancy, and it also leads to psychosocial consequences, including strained relationships between affected individuals and their caregivers or family members (Jaracz et al., 2002, Towfighi et al., 2017). Meanwhile, post-stroke fatigue refers to a condition characterized by a persistent sense of exhaustion and diminished energy levels experienced by individuals following a stroke event. A recent systematic review and meta-analysis revealed that post-stroke fatigue affects approximately 48 % of stroke survivors, yet it often receives insufficient attention from medical practitioners (Alghamdi et al., 2021). Assessment of fatigue often relies on subjective measurements and patients' self-reported experiences (Staub and Bogousslavsky, 2001). Post-stroke cognitive impairment refers to a range of deficits in cognitive functions, including memory, attention, language, and executive function, which manifest after a stroke. In Asia, several researchers have revealed the prevalence of post-stroke cognitive impairment ranges from 20 % to 40 % after 3 months (Zhou et al., 2005, Tu et al., 2014).

Rehabilitation treatment plays a crucial role in enabling stroke survivors to achieve optimal functional independence while minimizing or preventing impairments (Clare et al., 2013). However, this process can be both time-consuming and financially burdensome due to associated costs, such as medical procedures, ancillary or referral costs, and expenses incurred during recovery (Chongsuvivatwong et al., 2011). Therefore, in light of these challenges, individualizing exercises and promoting self-practice at home are recommended for post-stroke patients, particularly in low- and middle-income countries. Additionally, due to the limited availability of intensive inpatient rehabilitation programs in Vietnam, there is a growing demand for home-based rehabilitation (Anh et al., 2021). Previous publications have also indicated that family support and involvement positively influence long-term rehabilitation, with strong support from family members leading to better outcomes for stroke survivors (Young, 1994, Chaiyawat and Kulkantrakorn, 2012). Furthermore, there is still limited focus on early screening and the development of intervention strategies aimed at improving the mental well-being of post-stroke survivors in Vietnam. Previous researchers primarily focused on pharmacotherapies and behavioral interventions to improve mental symptoms (e.g., depression, fatigue, cognitive disorders) and enhance stroke recovery. These interventions include selective serotonin reuptake inhibitors, exercise, and music therapy (Sun et al., 2017, Qin et al., 2018). Despite their proven efficacy, safety, and integration into clinical practice, the optimal approach for preventing post-stroke mental health impairment in survivors remains uncertain (Ramasubbu, 2011, Lee et al., 2021).

Currently, Motivational Interviewing is considered a safer method compared to pharmacotherapies, using patient-centered and patient-oriented techniques aimed at motivating individuals to change their behavior (Arkowitz et al., 2015). Motivational Interviewing is a patient-centered collaborative technique aimed at encouraging behavior change and may offer a safer alternative to antidepressants (Arkowitz et al., 2015, Miller and Rollnick, 2004). Initially described in 1983 as a brief intervention for problem drinking, Motivational Interviewing has since been proven effective for various chronic health conditions, including hypertension, diabetes, cardiovascular disease, and obesity, where behavior modification is crucial (Rollnick et al., 2008). The first randomized controlled trial using an adapted version of Motivational Interviewing for acute stroke patients was conducted in the United Kingdom (Watkins et al., 2007)*.* While traditionally focused on behavior change, researchers in this study applied Motivational Interviewing to support psychological adjustment and emotional recovery following a stroke. Motivational Interviewing has been used across 35 nations and in 26 different languages, underscoring its widespread adoption and global popularity as a mental health intervention. Numerous meta-analyses have shown that Motivational Interviewing has positive effects on depression, fatigue, other mental symptoms, and treatment adherence among stroke patients within a follow-up period of less than 12 months (Qiqi et al., 2021, Potter et al., 2022).

In Vietnam, the National Geriatric Hospital annually admits a substantial cohort of stroke patients, who are managed and treated under the chronic disease management program. This program is designed to provide comprehensive care for these prevalent chronic diseases by implementing standardized protocols, with continuous support from the Vietnam Ministry of Health. The Vietnam National Geriatric Hospital plays a critical role in delivering both hospital-based and community-based care, ensuring that patients have access to coordinated, long-term management and follow-up services. Leveraging these resources, we aimed to evaluate a multi-intervention program for improving the physical and mental health of post-stroke patients. This study represents a crucial advancement in addressing the complex needs of post-stroke patients and expanding the field of intervention research in stroke care.

## 2 . Materials and methods

### 2.1 A randomized clinical trial

This was a randomized controlled trial conducted at the Vietnam National Geriatric Hospital to assess a multi-intervention approach for stroke survivors. Between December 2021 and March 2022, patients diagnosed with stroke by a neurologist were consecutively recruited to participate in the study. The study subjects were receiving either inpatient or outpatient treatment and were managed through the hospital's chronic disease management program.

Patients meeting the inclusion criteria were randomly assigned to either the intervention group, which received the long-term multi-intervention program, or the control group, which received standard care. The allocation of participants into the two groups was concealed from the study physician and the nurse who supported the intervention activities.

The randomization sequence was created using Stata 15.1 statistical software, stratified by age and sex, with a 1:1 allocation using random block sizes of four. This random sequence was produced by a staff member not involved in the trial. Each patient was assigned an anonymous study code, and a nurse, who was completely unrelated to the study, assisted in sending the anonymous code to participants through an envelope. Patients were assigned to the intervention or control group based on the corresponding code (A = intervention; B = control). This information was then provided to the principal investigator of the study.

Participants remained blinded to their group assignment. While the senior nurse in charge of data collection was not blinded, the statistician was unaware of the group assignments. Assessments were conducted at baseline and at 1, 3, and 6-month follow-up points to evaluate the outcomes of the intervention.

### 2.2 Inclusion and exclusion criteria

We selected patients who met the following inclusion criteria: (1) aged 45 years or older (as the study was conducted at the Central Geriatric Hospital in Vietnam, where the patient population primarily consists of individuals in this age group); (2) diagnosed with stroke according to the World Health Organization definition of stroke (WHO-MONICA-Project, 1988); (3) had a duration from stroke onset to study participation ranging from 1 month to 1 year; (4) were able to participate in intervention activities, such as Motivational Interviewing and rehabilitation treatments.

Stroke survivors who (1) were diagnosed with mental illnesses before the stroke occurrence (such as depressive disorder, anxiety disorder, emotional disorder, schizophrenia) or other neurological diseases; (2) experienced transient ischemic attacks or unstable medical conditions (e.g., consciousness disorders, coma, or limited communication); or (3) were unable to provide informed consent were excluded.

### 2.3 Sample size

We used the sample size formula for two means to calculate the sample size required for this study (comparing the intervention group with the control group after 6 months of intervention). The formula for calculating the sample size is as follows: *N* = *(Z_(1-α/2) +_ Z _(1-_*_β_*_)_)^2^/(μ_d_/σ_d_)^2^,* where *N* is the sample size required for each group; *Alpha* = 0.05; *Power* = 0.8; *μ_d_* = 3.43 and *σ_d_* = 5.49, determined according to a previous similar study of J.Sims et.al (Sims et al., 2009). The minimum sample size for each group was calculated to be 43, and the actual sample size obtained was 46 for each group. A total of 92 stroke patients were enrolled and observed for 6 months.

### 2.4 Intervention program

#### 2.4.1 Intervention group

##### 2.4.1.1 Motivational interviewing

Motivational Interviewing facilitators were primarily responsible for the implementation of this intervention. They were trained and completed the entire Motivational Interviewing in Healthcare course, which was guided by Motivational Interviewing trainers, including the principal investigator. The principal investigator had been granted a certificate of completion for continuing medical education in Motivational Interviewing in Healthcare (with License No. C0002141, issued by the Institute for Better Health in the United States). Additionally, the training program included two formal lessons for practice in simulated situations.

The fidelity assessment in this study comprised three main components: (1) audio recordings of sessions, (2) expert evaluations of competence, and (3) adherence to the study-specific Motivational Interviewing manual. Audio recordings were used to assess the fidelity of the intervention. Facilitators recorded all sessions with their assigned participant groups, ensuring prior consent for audio recording. The principal investigator selected the first session conducted by each facilitator for review. If a facilitator's intervention did not align with the principles outlined in the study-specific Motivational Interviewing manual, they received additional individualized training. Expert Motivational Interviewing trainers, responsible for both training and supervision throughout the trial, conducted the competence assessments. They reviewed the full audio recordings and rated Motivational Interviewing competence using a four-point scale (1 = inconsistent, 2 = below beginning proficiency, 3 = beginning proficiency, 4 = competent). The assessment focused on three key dimensions: (i) the facilitator's overall Motivational Interviewing competence during the session, (ii) their embodiment of the Motivational Interviewing spirit (e.g., partnership, empathy, and support for participant autonomy), and (iii) their ability to engage participants through rapport-building, collaboration, and eliciting change talk. Adherence to the Motivational Interviewing manual was evaluated using a five-point Likert scale (0 = not at all, 1 = to a small degree, 2 = to some degree, 3 = to a large degree, 4 = to a very large degree). Facilitators' adherence to the manual's key themes was scored based on audio recordings, with evaluations conducted by the trainers. The scores were then discussed in a consensus meeting to ensure consistency in the ratings. Facilitators who did not meet the required standards received additional individualized training.

Initially, four nurses and two social workers volunteered for this program. There were two main reasons why nurses and social workers were chosen to conduct Motivational Interviewing therapy. First, their ability to empathize with patients in this context is more important than having specialized knowledge of Motivational Interviewing (Lundahl and Burke, 2009). Second, if Motivational Interviewing proves beneficial for stroke patients, involving these professionals can help ensure its long-term implementation and sustainability. This is because study participants were invited to participate while also receiving treatment (inpatient or outpatient) and were managed according to the hospital's program, where nurses primarily supported the study context. Following randomization, facilitators conducted face-to-face (1:1) Motivational Interviewing therapy sessions with participants. Facilitator selection was based on their availability, ensuring they were scheduled for subsequent appointments to administer the therapy. Facilitators were solely responsible for delivering Motivational Interviewing therapy and did not participate in the recruitment process.

The overarching principle of the intervention was to support stroke survivors to adjust to life after stroke. The intervention covered three key components. The first content focused on setting the agenda and encouraging the patient to talk about stroke adjustment. The second component involved helping the patient identify realistic recovery goals and barriers to achieving those goals. The third component centered on exploring any ambivalence the patient might have about achieving goals, supporting the patient's optimism and self-efficacy, and assisting in identifying solutions to solve problems. Goals, commitments, and relevant information from participants were encouraged to be summarized and clarified from the first two sessions. The intervention consisted of eight sessions in total: once a week during the first month, followed by sessions every 2 weeks during the second and third months. Each appointment lasted approximately 1 hour. The sessions were conducted at the Central Geriatric Hospital in Vietnam, where patients were scheduled and invited into a private room. This setting ensured both confidentiality and a focused, supportive environment for the Motivational Interviewing sessions.

##### 2.4.1.2 Home-based rehabilitation program

A home-based individual exercise program was implemented and guided by physical therapists. Physical therapists provided guidance to patients in six separate sessions and conducted home-based rehabilitation visits: once a week during months 1 and 2, every 2 weeks during months 3 and 4, and every 4 weeks during months 5 and 6. The physical therapists' phone numbers were given to patients and their caregivers for consultation regarding the home-based rehabilitation program. Daily records were used to monitor compliance throughout the intervention, regardless of whether compliance was high or low. Various functions, including basic activities of daily living, as well as indoor and outdoor mobility, were assessed before establishing the individual home-based rehabilitation program for participants. If needed, individual counseling was provided to the caregivers, focusing on education, practical application of information, and addressing problems occurring at home.

The rehabilitation strategy was designed based on the principles of motor learning, the mirror neuron concept, and exercise physiology (Barnes, 2003, Buccino et al., 2006, Brandstater, 1990, Jurkiewicz et al., 2011). The individualized program was developed through the combined input of experts and various therapists including physical, occupational, and speech therapists, as well as stroke patients. The strategy included an audiovisual compact disc with standard rehabilitation procedures such as passive, active, and resistance exercises, along with activities of daily living. These activities included tasks such as using a lock and key, preparing a drink, using a cane or wheelchair, and putting on and taking off shoes. The therapist documented the duration and type of therapy on a case report form. The prescribed time for each rehabilitation session at home was 1 hour. Patients or their caregivers were asked to maintain diaries detailing the duration and nature of rehabilitation sessions. Caregivers received instructions on assisting patients in ways that encouraged the use of their functional abilities as much as possible. Assessments were conducted at the start and then at 1-, 3-, and 6-month intervals.

##### 2.4.1.3 Periodic health check program

Participants were assessed periodically for their health, including physical and mental health, recurrence risks, and harmful behaviors at 0, 1, 3, and 6 months. At the Central Geriatric Hospital in Vietnam, doctors supported the periodic health assessments of participants through the outpatient treatment program and managed co-morbid chronic diseases, such as hypertension, diabetes, and chronic cardiovascular disease.

#### 2.4.2 Control group

The control group received a periodic health check program similar to the intervention group. In cases where participants were diagnosed with any mental disorders, they were referred for psychiatric assessment and provided with professional stroke handouts, such as those issued by the Vietnam Ministry of Health, during the study period. While the intervention group received structured, guided sessions and follow-up home visits from physical therapists, the control group was given general written instructions for home-based rehabilitation, without any therapist-guided sessions or follow-up visits. Any additional treatments were documented in the case report form.

### 2.5 Data collection and instruments

Data were gathered at four intervals: baseline, 1-month, 3-month, and 6-month follow-up points.

#### 2.5.1 Basic demographic data and stroke-related characteristics

*Demographic characteristics:* age, sex (male/female).

*Stroke-related characteristics:* These variables were collected based on several aspects: (i) Stroke classification (ischemia/hemorrhage/unknown), derived from medical records; (ii) Frequency of stroke occurrences (once/twice or more); (iii) Duration from stroke onset to participation in the study (1 month to < 3 months/3 months to < 6 months/6 months to 1 year); (iv) Hemispheric lesion locations (left-sided/right-sided/unknown); (v) Disability levels of stroke survivors according to the Modified Rankin Scale (0 - no symptoms/1 - no significant disability/2 - slight disability/3 - moderate disability/4 - moderate-severe disability/5 - severe disability); (vi) National Institutes of Health Stroke Scale score (Kwah and Diong, 2014).

#### 2.5.2 Primary outcome: mental health change outcome variables

*Post-stroke Depression:* The Patient Health Questionnaire-9 is part of the Primary Care Evaluation of Mental Disorders and serves as a diagnostic tool for screening and assessing depression severity. It functions as a self-administered questionnaire (Kroenke et al., 2001). The Patient Health Questionnaire-9 consists of nine questions, with each item rated on a scale from "0″ (not at all) to "3″ (nearly every day), resulting in a cumulative score of up to 27. The Vietnamese version was tested for validity and reliability in assessing depressive symptoms (Vu et al., 2022).

*Post-stroke Fatigue:* The Fatigue Severity Scale is frequently used to evaluate the effect of fatigue on various aspects of daily life, particularly in post-stroke survivors (Ozyemisci-Taskiran et al., 2019). The Fatigue Severity Scale comprises ten items, with each item rated on a scale from 1 (strongly disagree) to 7 (strongly agree), resulting in an overall score range of 0 to 70. Higher Fatigue Severity Scale scores indicate increased fatigue severity. In the present study, Cronbach's alpha coefficients for the item responses demonstrated high internal consistency, measuring at 0.97, 0.98, 0.99, and 0.99 across the four successive follow-up assessments.

*Cognitive Impairment:* The Mini-Mental State Examination consists of 11 items assessing various cognitive domains, including orientation to time and place (2 items), registration (1 item, where the individual repeats a set of objects), attention and calculation (1 item), recall (1 item), language (1 item), repetition (1 item), and complex commands (4 items). With a total score of 30, participants are considered to have cognitive impairment if they score 24 or lower (Bour et al., 2010). The scale had previously been validated for screening cognitive impairment in the Vietnamese community (Long et al., 2022).

#### 2.5.3 Primary outcome: physical health change outcome variables

*Activities of Daily Living:* The Barthel Index assesses an individual's level of independence and mobility in performing daily activities such as feeding, bathing, grooming, dressing, controlling bowel and bladder functions, using the toilet, transferring from a seated position, walking, and climbing stairs. With a score range of 0 to 100, higher scores indicate greater levels of independence in daily activities. This scale had been validated in Vietnamese populations in previous research (Takahashi et al., 2011).

#### 2.5.4 Secondary outcome: quality of life

*Stroke Impact Scale 3.0:* This scale evaluates the post-stroke quality of life, utilizing a scoring system ranging from 0 to 100, where higher scores denote improved quality of life (Duncan et al., 2003). It comprises eight domains: strength, hand function, mobility, instrumental activities of daily living, memory and thinking, communication, emotion, and social participation.

All assessments, including the cognitive impairment evaluation using the Mini-Mental State Examination, were conducted by nurses trained by a neuropsychologist. The nurses were also trained by a psychiatrist and a physical therapist to administer other questionnaires, including the Patient Health Questionnaire-9, Fatigue Severity Scale, Barthel Index, and Stroke Impact Scale 3.0.

### 2.6 Statistical analysis

STATA Statistical Software (version 16.0) and RStudio were used to process and analyze the current dataset. Continuous variables were expressed as the means and standard deviations (SD), while categorical variables were presented as frequencies and percentages. The Chi-squared test was utilized for categorical variables, whereas the Wilcoxon rank-sum test was employed for continuous variables to detect statistically significant differences between the two groups.

The independent samples *t*-test was utilized to compare the mean differences of dependent variables (Patient Health Questionnaire-9, Fatigue Severity Scale, Mini-Mental State Examination, Barthel Index, and Stroke Impact Scale 3.0) between the two groups at various time points. Additionally, variables with multiple measurements over time were compared using the repeated measures Analysis of Variance (ANOVA) test to assess effectiveness before and after intervention in two groups, calculating the time*group interaction (Singh et al., 2013). Moreover, effect sizes after intervention were measured using Cohen's *d*, with values indicating small (d = 0.2), medium (d = 0.5), and large (d ≥ 0.8) effects (Cohen, 2013).

### 2.7 Ethics approval

**Fig. 1:**
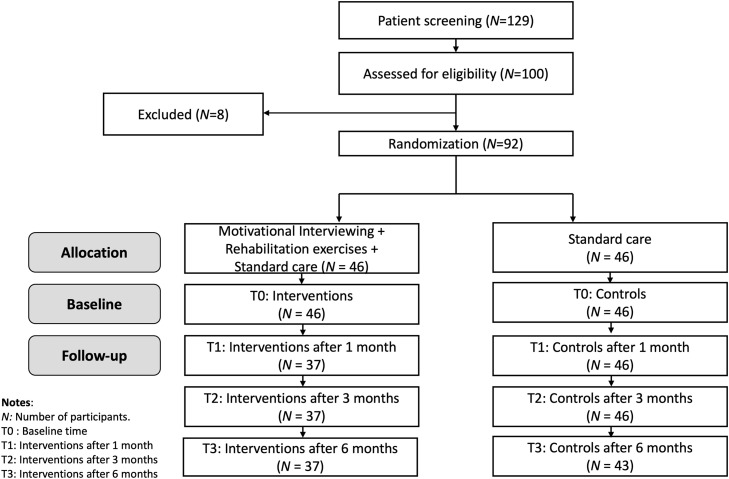
CONSORT flow diagram.

The study obtained approval from the Institutional Review Board for Ethics in Biomedical Research at Hanoi Medical University (Approval Code: No. 494/GCN-HDDDNCSYHN-DHYHN, dated 05/12/2021). Before participating in the study, all participants or their respective relatives/guardians provided written informed consent in accordance with the Declaration of Helsinki. Fig. 1

## 3 Results

### 3.1 Participant flow and recruitment

Out of 129 stroke patients invited, 100 (77.5 %) agreed to participate, with 92 meeting the inclusion criteria. These participants were randomly assigned to the intervention (46) and control (46) groups. Over 6 months, 37 participants in the intervention group completed the program, which involved home-based rehabilitation and Motivational Interviewing. The intervention group had a lower dropout rate (6.5 %) compared to the control group (17.4 %). Reasons for dropout included medical issues, loss to follow-up, and inconvenience. Baseline data for the study were collected from December 2021 to March 2022, with the primary completion date being January 30, 2023.

### 3.2 Baseline data

Table 1 displays the demographic characteristics of the intervention and control groups. Except for age, hemispheric lesion locations, and National Institutes of Health Stroke Scale scores, no statistically significant differences were found between the groups. Both groups had an equal proportion of women. Respondents reported various stroke statuses, with ischemia being the most common. The majority of post-stroke patients had experienced only one stroke and participated in the study between 3 to 6 months after stroke onset. Most participants were classified in the moderate-to-severe disability category according to the Modified Rankin Scale, with intervention group patients showing lower National Institutes of Health Stroke Scale scores compared to the control group.

**Table 1:** . Demographic characteristics and stroke characteristics of participants.

Variables	Internvention		Control		Total		*p*-value
	(*n* = 46)		(*n* = 46)		(*n* = 92)		
	*n*	%	*n*	%	*n*	%	
Sex							
Male	24	52.2	24	52.2	48	52.2	1.000
Female	22	47.8	22	47.8	44	47.8	
Stroke classification							
Ischemia	21	45.7	15	32.6	36	39.1	0.428
Hemorrhage	5	10.9	7	15.2	12	13.0	
Unknown	20	43.5	24	52.2	44	47.8	
Frequency of stroke occurrences							
Once	34	73.9	31	67.4	65	70.7	0.492
Twice or more	12	26.1	15	32.6	27	29.3	
Duration from stroke onset to participate in the study							
1 month to < 3 months	9	19.6	11	23.9	20	21.7	0.798
3 months to < 6 months	22	47.8	19	41.3	41	44.6	
6 months to 1 year	15	32.6	16	34.8	31	33.7	
Hemispheric lesion locations							
Left-sided	6	13.0	0	0.0	6	6.5	0.027
Right-sided	17	37.0	24	52.2	41	44.6	
Unknown	23	50.0	22	47.8	45	48.9	
Disability levels of stroke survivors - Modified Rankin Scale							
No symptoms (0)	5	10.9	1	2.2	6	6.5	0.353
No significant disability (1)	9	19.6	12	26.1	21	22.8	
Slight disability (2)	5	10.9	4	8.7	9	9.8	
Moderate disability (3)	9	19.6	6	13.0	15	16.3	
Moderate-severe disability (4)	18	39.1	23	50.0	41	44.6	
	Mean	SD	Mean	SD	Mean	SD	*p*-value
Age	74.7	10.2	69.8	12.1	72.2	11.4	0.048
National Institutes of Health Stroke Scale (score)	5.9	4.6	9.2	6.4	7.6	5.8	0.010

SD: Standard deviation.

Overall, the mean scores of mental health assessment scales (Patient Health Questionnaire-9, Fatigue Severity Scale, and Mini-Mental State Examination) were similar between the two groups at T0. Likewise, the mean score for the Barthel Index at baseline (T0) was also comparable between the intervention and control groups. However, the intervention group had lower Stroke Impact Scale 3.0 scores compared to the control group (Table 2).

**Table 2:** Baseline physical, mental health and wellbeing characteristics of both groups.

Variables	Internvention (*n* = 46) Mean ± SD	Control (*n* = 46) Mean ± SD	Total (*n* = 92) Mean ± SD	Mean difference 95 %CI, *p*-value*	Effect size^⁎⁎^
Patient Health Questionnaire-9 T0 (score 0 - 27)	10.9 ± 6.5	10.3 ± 5.9	10.6 ± 6.2	0.6 (-1.9; 3.2), *p* = 0.617	0.10
Fatigue Severity Scale T0 (score 0–70)	35.2 ± 12.9	37.8 ± 11.4	36.5 ± 12.2	-2.6 (-7.6; 2.5), *p* = 0.323	-0.21
Mini-Mental State Examination T0 (score 0–30)	16.2 ± 7.0	18.3 ± 8.9	17.3 ± 8.05	-2.1 (-5*.*4; 1.3), *p* = 0.221	-0.26
Barthel Index T0 (score 0–100)	55.5 ± 36.2	55.4 ± 34.3	55.5 ± 35.1	0.1 (-14.5; 14.7), *p* = 0.988	0.03
Stroke Impact Scale 3.0 T0 (score 0–100)	45.4 ± 16.4	53.2 ± 19.2	49.3 ± 18.2	-7.8 (-15.2; -0.3), *p* = 0.040	-0.43

*The independent samples *t*-test is used to compare the mean difference between the two groups at time points.

**Effect Sizes are measured by Cohen's d.

T0: Baseline time; SD: Standard deviation.

### 3.3 Post-intervention

The intervention group showed significant improvements in mental health, with reductions in Patient Health Questionnaire-9 scores, indicating reduced depressive symptoms. Fatigue levels, as measured by the Fatigue Severity Scale, also decreased significantly. Cognitive function, assessed by the Mini-Mental State Examination, showed moderate improvement. Physical function, measured by the Barthel Index and Stroke Impact Scale 3.0 scores indicated substantial improvement over time. The repeated measures ANOVA confirmed the effectiveness of the interventions across all metrics, as detailed in Table 3.

**Table 3:** Physical, mental health and wellbeing characteristics at 1 month (T1), 3 months (T2), 6 months (T3) follow-up.

Variables		Internvention (*n* = 37) Mean ± SD	Control (*n* = 46) Mean ± SD	Between-group comparisons		Repeated measures ANOVA test, *p*-value⁎⁎⁎
				Mean difference 95 %CI, *p*-value* (each time points)	Effect size⁎⁎	
Patient Health Questionnaire-9	T1	9.1 ± 1.0	19.7 ± 3.8	-10.6 (-12.7; -8.4), *p* = 0.000	-2.16	F = 22.53 *p* = 0.000
	T2	3.1 ± 2.8	24.5 ± 2.8	-21.4 (-22.5; -20.1), *p* = 0.000	-7.67	
	T3	1.8 ± 1.9	25.6 ± 2.9	-23.8 (-25.0; -22.8), *p* = 0.000	-9.78	
Fatigue Severity Scale	T1	28.5 ± 11.8	43.4 ± 8.7	-14.9 (-19.4; -10.4), *p* = 0.000	-1.45	F = 24.21 *p* = 0.000
	T2	17.8 ± 5.9	53.9 ± 8.1	-36.1 (-39.9; -33.6), *p* = 0.000	-5.11	
	T3	14.4 ± 4.9	57.9 ± 7.6	-43.5 (-46.4; -40.7), *p* = 0.000	-6.72	
Mini-Mental State Examination	T1	13.5 ± 9.7	18.5 ± 8.7	-5.0 (-8.8; -1.1), *p* = 0.012	-0.53	F = 16.45 *p* = 0.000
	T2	17.9 ± 10.3	16.2 ± 7.6	1.7 (-2.1; 5.4), *p* = 0.389	0.18	
	T3	19.5 ± 10.6	15.0 ± 8.3	4.5 (0.6; 8.4), *p* = 0.026	0.47	
Barthel Index	T1	58.8 ± 35.2	55.7 ± 33.9	3.1 (-12.0; 18.3), *p* = 0.682	0.09	F = 60.47 *p* = 0.000
	T2	68.8 ± 28.6	56.2 ± 33.3	12.6 (-1.2; 26.3), *p* = 0.072	0.40	
	T3	68.8 ± 28.6	54.5 ± 33.0	14.3 (0.4; 28.1), *p* = 0.044	0.46	
Stroke Impact Scale 3.0	T1	48.3 ± 15.3	44.7 ± 19.6	3.6 (-4.2; 11.4), *p* = 0.364	0.20	F = 40.92 *p* = 0.000
	T2	58.5 ± 15.1	40.5 ± 19.2	18.0 (10.3; 25.7), *p* = 0.000	1.03	
	T3	62.6 ± 13.8	37.0 ± 18.8	25.6 (18.2; 33.1), *p* = 0.000	1.54	

⁎ The independent samples *t*-test is used to compare the mean difference between the two groups at time points.

⁎⁎ Effect Sizes are measured by Cohen's d.

⁎⁎⁎ Repeated Measures Analysis of Variance (ANOVA) test compared effectiveness before and after intervention in two groups, calculating time*group interaction. T1: Interventions after 1 month; T2: Interventions after 3 months; T3: Interventions after 6 months; SD: Standard deviation.

## 4 Discussion

This study was the first randomized controlled trial to assess the intervention's impact on both physical and mental health among post-stroke survivors in Vietnam. The intervention group demonstrated substantial improvements across mental and physical health areas after receiving a multifaceted intervention, including motivational interviewing and a home-based rehabilitation program. Notably, depression and fatigue levels, as measured by the Patient Health Questionnaire-9 and Fatigue Severity Scale scores, decreased significantly, indicating strong improvements in these two metrics. Cognitive function, assessed via the Mini-Mental State Examination, showed moderate improvement, while physical capabilities, evaluated by the Barthel Index, improved significantly after 3 and 6 months during the study period.

The intervention group demonstrated significant improvements across several measurements, showing both statistical and clinical significance. The Patient Health Questionnaire-9 scores showed a significant reduction in depressive symptoms over time. This reduction was not only statistically significant but also clinically meaningful, as it reflects a transition from mild depression to minimal or no depression, likely leading to improvements in mental health and quality of life. Similarly, fatigue levels, as measured by the Fatigue Severity Scale, showed a substantial reduction, suggesting a substantial reduction in fatigue that is likely to improve patients' daily energy levels and ability to function. The implementation of Motivational Interviewing techniques appears to have led to notable enhancements in both depression and fatigue levels among the participants. From these findings, we suggest that the tailored approach of Motivational Interviewing, which emphasizes empathetic communication and goal-oriented strategies, holds promise in addressing the complex psychological and physical challenges faced by stroke survivors. Previous researchers on Motivational Interviewing have demonstrated noteworthy reductions in depressed mood and mortality rates observed at 12 months post-stroke (Watkins et al., 2011). In this study, depression and fatigue were found to decline over time. This outcome might be attributed to the timing and intensity of the intervention, as we administered eight Motivational Interviewing sessions spread across 3 months. This finding parallels that of a previous study, although differing in terms of the timing and intensity, wherein four Motivational Interviewing sessions spaced over 9 months were mentioned in similar research (Barker-Collo et al., 2015). In a prior study, researchers implemented weekly sessions for up to 4 weeks, beginning within 4 weeks post-stroke, and showed comparable outcomes (Watkins et al., 2011). A meta-analysis indicated that Motivational Interviewing may contribute to improving depressive symptoms, particularly highlighting its significant impact on depression levels during follow-up periods of less than 12 months. Conversely, no notable benefits were discerned after the 12-month follow-up (Qiqi et al., 2021). Moreover, Motivational Interviewing could potentially serve as an influential component within a fatigue-specific intervention program, enhancing the motivation and determination of stroke survivors to move forward (Watkins et al., 2007).

We showed improvement in functional independence (Barthel Index) after participants received a multi-intervention, which included Motivational Interviewing therapy. Earlier researchers have suggested that the Motivational Interviewing approach may enhance Barthel Index scores. However, patients still require ongoing physical and functional therapy to promote their reintegration into daily life and effectively increase their activities of daily living (Chen et al., 2020). Typically, individuals who have experienced their first stroke may suffer psychological impacts. Researchers have indicated that approximately one third of stroke survivors were affected by post-stroke mental health issues, such as depression, fatigue, or cognitive impairment, after their initial stroke (Blomgren et al., 2019, Hackett and Pickles, 2014). The application of Motivational Interviewing techniques demonstrated a significant reduction in both depression and fatigue symptoms, indicating its potential effectiveness as an intervention method in this population. However, additional research with larger sample sizes and longer follow-up periods should be conducted to corroborate these findings and ascertain the sustained benefits of Motivational Interviewing in this context. Furthermore, future research exploring potential mediators and moderators of Motivational Interviewing's effects could provide valuable insights into its mechanisms of action and optimize its implementation in clinical practice for post-stroke rehabilitation.

We have elucidated the remarkable advantages and benefits of a home-based rehabilitation program through substantial evidence. Physical function, measured by the Barthel Index, improved significantly, indicating a meaningful increase in independence in activities of daily living, which is clinically relevant as it enhances patients’ autonomy and quality of life. Over 6 months, the home-based exercise program resulted in greater improvements and higher levels of functional independence in the intervention group. Additionally, this intervention appeared to accelerate recovery within the first 3 months compared to usual care. After 3 months, the benefits of this intervention showed slight improvement and reached a plateau by 6 months. Key factors contributing to these results included family support, a home-based rehabilitation environment, an individualized program with audiovisual materials, and close follow-up (Chaiyawat and Kulkantrakorn, 2012, Langhorne et al., 2011). Most patients and caregivers favored home-based rehabilitation because it allowed them to remain close to their families. This may indicate the strong ties within extended families, which could enhance their support capabilities (Heinemann et al., 1987). Previous researchers have also shown functional improvement with 6–8 hours per day of constraint-induced exercise (Liepert et al., 2000). In contrast, our protocol involved only 1 hour per day, with encouragement for independent practice. However, we also supplied audiovisual materials, which helped support their progress through an intensive, motivating, and progressive program. Utilizing rehabilitation exercises, especially focusing on individualizing exercises and promoting self-practice at home combined with Motivational Interviewing, resulted in relatively positive changes in the intervention group compared to controls. By tailoring exercises to each individual's abilities and needs, patients can engage in activities that are both feasible and beneficial for their recovery. Researchers have also determined that individualizing exercises combined with Motivational Interviewing help facilitate goal-setting and shared decision-making in stroke rehabilitation (Kim et al., 2023, Chen et al., 2020). Motivational Interviewing was also confirmed to help enhance motivation for rehabilitation among stroke patients, particularly when combined with home exercise programs. This underscores the efficacy of Motivational Interviewing as a targeted approach to increase the intrinsic motivation of stroke patients (Chen et al., 2020). This combination not only improves physical functioning but also enhances overall well-being by instilling motivation in stroke survivors, leading to better outcomes in their recovery process.

## 5 Limitations

Our study had several limitations, which reduced its value and relevance. Firstly, the small sample size limited the thorough investigation of underlying mechanisms and mediating factors. Additional research with larger sample sizes and longer follow-up periods should be conducted to corroborate these findings and ascertain the sustained benefits of interventions in this study. Secondly, participant heterogeneity, including variations in stroke timing, depression onset, received rehabilitation, severity, comorbidities, and medications, may have confounded the results. Future studies could employ more detailed recruitment strategies to address these complexities. Thirdly, all participants in the intervention group had family support, even though this was not a selection criterion. While this fortunate circumstance may have contributed to the overall success of the intervention, it limits our ability to compare outcomes for participants without caregiver support. Future studies should specifically consider including participants who lack family or caregiver assistance to provide a more comprehensive understanding of the role of support systems in home-based rehabilitation programs. Fourthly, as the intervention group received more attention and support compared to the standard care control group, it is not possible to definitively determine whether the improvements in recovery, depression, and fatigue were solely due to the Motivational Interviewing techniques and home-based rehabilitation program or whether they were influenced by the additional attention given to the group. Future studies should consider including an attention control arm to isolate the effects of the intervention itself from the potential benefits of increased attention. Lastly, the 6-month follow-up may not capture the long-term effects of the intervention, given the chronic nature of post-stroke mental and physical health impairment. Further investigation is needed to assess the sustainability of short-term benefits from Motivational Interviewing and early rehabilitation.

## 6 Conclusion

We aimed to evaluate the impact of a multi-intervention program on the mental and physical health of stroke survivors with chronic sequelae. We found that participants who underwent Motivational Interviewing therapy and home-based rehabilitation were more likely to experience substantial improvements across mental and physical health metrics. Additional research will be necessary to precisely identify which characteristics of stroke survivors are most conducive to benefiting from the current intervention program. The findings highlight the importance of healthcare providers addressing both mental health recovery and physical rehabilitation, with a particular focus on home-based services for stroke survivors.

## CRediT authorship contribution statement

**Thao Thi Phuong Nguyen:** Writing – review & editing, Writing – original draft, Supervision, Project administration, Methodology, Investigation, Formal analysis, Data curation, Conceptualization. **Hai Bui Hoang:** Writing – review & editing, Supervision, Methodology, Formal analysis, Conceptualization. **Huyen Thi Thanh Vu:** Writing – review & editing, Supervision, Methodology, Formal analysis, Conceptualization.

## Declaration of competing interest

The authors declare that they have no known competing financial interests or personal relationships that could have appeared to influence the work reported in this paper.
